# The Gene Ontology of eukaryotic cilia and flagella

**DOI:** 10.1186/s13630-017-0054-8

**Published:** 2017-11-16

**Authors:** Paola Roncaglia, Teunis J. P. van Dam, Karen R. Christie, Lora Nacheva, Grischa Toedt, Martijn A. Huynen, Rachael P. Huntley, Toby J. Gibson, Jane Lomax

**Affiliations:** 1 European Molecular Biology Laboratory, European Bioinformatics Institute (EMBL-EBI), Wellcome Genome Campus, Hinxton, Cambridge, CB10 1SD UK; 2 The Gene Ontology Consortium, http://geneontology.org; 3 Centre for Molecular and Biomolecular Informatics, Radboud University Medical Center, PO Box 9101, 6500 HB Nijmegen, The Netherlands; 4 Theoretical Biology and Bioinformatics, Department of Biology, Faculty of Science, Utrecht University, Utrecht, The Netherlands; 5 The Jackson Laboratory, 600 Main Street, Bar Harbor, ME 04609 USA; 6 Fakultät Biowissenschaften, Universität Heidelberg, Im Neuenheimer Feld 234, 69120 Heidelberg, Germany; 7 Structural and Computational Biology Unit, European Molecular Biology Laboratory, Meyerhofstr. 1, 69117 Heidelberg, Germany; 8 Present Address: Centre for Cardiovascular Genetics, University College London, London, WC1E 6JF UK; 9 Present Address: SciBite Limited, BioData Innovation Centre, Wellcome Genome Campus, Cambridge, CB10 1DR UK

**Keywords:** Cilium, Flagellum, Gene Ontology, Ciliopathy, Basal body, Axoneme, Microscopy image annotation, Systems biology

## Abstract

**Background:**

Recent research into ciliary structure and function provides important insights into inherited diseases termed ciliopathies and other cilia-related disorders. This wealth of knowledge needs to be translated into a computational representation to be fully exploitable by the research community. To this end, members of the Gene Ontology (GO) and SYSCILIA Consortia have worked together to improve representation of ciliary substructures and processes in GO.

**Methods:**

Members of the SYSCILIA and Gene Ontology Consortia suggested additions and changes to GO, to reflect new knowledge in the field. The project initially aimed to improve coverage of ciliary parts, and was then broadened to cilia-related biological processes. Discussions were documented in a public tracker. We engaged the broader cilia community via direct consultation and by referring to the literature. Ontology updates were implemented via ontology editing tools.

**Results:**

So far, we have created or modified 127 GO terms representing parts and processes related to eukaryotic cilia/flagella or prokaryotic flagella. A growing number of biological pathways are known to involve cilia, and we continue to incorporate this knowledge in GO. The resulting expansion in GO allows more precise representation of experimentally derived knowledge, and SYSCILIA and GO biocurators have created 199 annotations to 50 human ciliary proteins. The revised ontology was also used to curate mouse proteins in a collaborative project. The revised GO and annotations, used in comparative ‘before and after’ analyses of representative ciliary datasets, improve enrichment results significantly.

**Conclusions:**

Our work has resulted in a broader and deeper coverage of ciliary composition and function. These improvements in ontology and protein annotation will benefit all users of GO enrichment analysis tools, as well as the ciliary research community, in areas ranging from microscopy image annotation to interpretation of high-throughput studies. We welcome feedback to further enhance the representation of cilia biology in GO.

**Electronic supplementary material:**

The online version of this article (10.1186/s13630-017-0054-8) contains supplementary material, which is available to authorized users.

## Background

The lens-making skills of Antonie van Leeuwenhoek provided him with the highest magnification microscopes that had yet to be made. With these tools, in a 1676 letter to the Royal Society, he reported the existence of protozoa, also describing their beating cilia and flagella [1]. That these two organelles are homologous to each other became clear when Irene Manton used electron microscopy to reveal the typical 9 + 2 arrangement of the microtubule doublets in motile axonemes [2]. However, the full biomedical significance of these organelles has only begun to be established with the realization that non-motile primary cilia of vertebrates are the site of many critical signalling pathways, notably for sonic hedgehog which plays key roles in embryonic development [3], as well as being sensory devices for many of our basic senses [4]. Thereafter, cilia research rapidly entered the era of ciliopathies-inherited diseases involving defects in cilia, gaining intense interest from human geneticists in addition to the broader biological research areas in which these organelles play key roles [5, 6] (see Additional file 1).

However, primary cilia were often dismissed as the “cell’s appendix”, rarely discussed in textbooks or research papers, and even more rarely depicted in diagrams of the numerous types of differentiated cell types that possess them; many aspects of cilia biology remain poorly understood. In addition, much of the older knowledge is not available electronically and therefore not accessible to be applied in modern disease discovery programmes, which typically use whole genome approaches to link candidate mutations to gene functional annotation.

One of the indispensable resources for function annotation used in genome research is the Gene Ontology (GO). The GO is a computational representation of biological knowledge that defines concepts used to describe aspects of gene function, and the relationships between these concepts. It consists of three main branches: Molecular Function (e.g. ‘ciliary neurotrophic factor receptor activity’), Biological Process (e.g. ‘ciliary transition zone assembly’) and Cellular Component (e.g. ‘ciliary transition zone’). Biocurators can then associate GO terms with specific gene products (proteins and RNAs) to capture experimental findings from the scientific literature [7, 8]; these associations are known as GO annotations. GO annotations are used widely by researchers as a way to generate hypotheses from data, in particular via enrichment analysis. For example, the PANTHER online resource [9] hosts a tool to perform GO enrichment analysis on user-defined gene sets, to help identify the biological processes or cellular components enriched in the set. Using this type of approach, the role of the DNA-binding protein RFX2 in spermatogenesis has been assessed and confirmed [10], while specific ciliary functions were shown to be present in the ampulla and isthmus of the bovine oviduct [11]. A well-structured GO representation of the cilium and cilium-mediated processes greatly affects the ability to capture information from the literature, and hence the quality of the outcomes of data analyses. Furthermore, the more fine-grained the representation, the more informative, insightful and useful a GO enrichment analysis can be. This is especially true for the cilium, where a gene product’s compartmentalization and biological process can be quite restricted and highly specific. For instance, many proteins involved in ciliopathies are located in particular ciliary substructures, such as the transition zone for Meckel-Gruber and Joubert syndromes [12] and the BBSome complex for Bardet-Biedl syndrome [13]. GO annotations form a knowledgebase, reflecting the collected information from a vast body of literature. The knowledge capture of ciliary protein functions and subcellular localizations will be even more relevant as new disorders are classified as ciliopathies [14]. As such, GO is indispensable when studying the cilium from a systems biology perspective.

Until a decade ago, the cilium was a little-appreciated organelle in the vertebrate cell, and the paucity of information in the literature was mirrored by a limited number of corresponding concepts and annotations in the Gene Ontology. Due to the importance of GO in providing cellular functional and contextual information for large-scale genomic and proteomic analyses, ciliary factors were effectively excluded from many contemporary systematic surveys of the cell. Then, more recently, an increasing focus on ciliary research highlighted the need to improve representation and capture of cilia-related knowledge in GO. Some of this knowledge has been included in the SysCilia standard (SCGS) database that captures known human cilium genes in a relatively simple list with genes and their location in the cilium [15]. In this article, we report on the steps we have taken towards a major revision of ciliary component and process terms in GO, and on the curation of human ciliary proteins that was made possible by such revision.

## Methods

### Ontology development

Members of the SYSCILIA Consortium [16] contacted the Gene Ontology Consortium (GOC) editorial team to discuss the need for a more complete and up-to-date formal representation of ciliary composition and biology. A team at Mouse Genome Informatics had also begun a project focused on annotation of mouse ciliary proteins and encountered the need for additional GO development in this area (Christie and Blake [17]). A working group was formed involving GO editors, GO biocurators and members of SYSCILIA. Engagement of the larger cilia research community was ensured in multiple ways, including communicating with SYSCILIA and other researchers and referring to a broad corpus of literature. Opinions outside the working group were especially sought in debatable cases.

SYSCILIA provided an initial list of suggestions for new terms to be added in GO, as well as changes to existing terms. Initially, the scope of the work was restricted to ciliary subcellular components, but as curation of relevant literature progressed, the effort was soon broadened to cover cilia-related biological processes as well. To record discussions on ontology development, and to allow members of GO and SYSCILIA outside the working group to contribute, we used a public tracker on the GitHub GO repository, specifically devoted to ontology requests [18]. The outcome of such discussions was the incorporation of new classes (terms) in GO, or the modification of existing classes. The modifications ranged from simple changes, such as the addition of a synonym, to more complex ones, such as creating links with other ontology classes. GO editors then implemented these additions and changes manually via the ontology editing tools Protégé [19] or OBO-Edit [20]. Also, some pattern-based classes (mostly to represent regulation of ciliary processes and localization to ciliary components) were added using an automated generator of GO terms called TermGenie [21].

### Annotation procedure

Human ciliary proteins were manually associated with GO terms according to recommended GO annotation procedures [22]. Annotation is performed by GO biocurators, who read relevant scientific articles and associate gene products with GO classes based on experimental evidence. The resulting annotations consist of a protein identifier, a GO term, an evidence code (based on the type of knowledge available, see [23]), and a reference to the scientific literature (mostly via a PubMed identifier). Where appropriate, the expressivity of annotations was increased by capturing information related to cell types such as ‘respiratory epithelial cell’ (by referring to the Cell Ontology term CL:0002368), or anatomical locations such as ‘trachea’ (using the Uberon anatomy term UBERON:0003126), as detailed in [24]. The Protein2GO tool provided by EMBL-EBI was used to associate gene products with GO classes [25]. As part of this ciliary curation effort, human proteins from the SYSCILIA Gold Standard set [15] were annotated to both ciliary and non-ciliary GO terms, to fully capture the experimental information provided. Where the same literature provided knowledge about ciliary genes from other species (e.g. rat or mouse), these genes were also annotated.

### Term enrichment analysis

Two versions of GO were downloaded from the Gene Ontology Consortium archive ftp-server (2012-12-01 and 2017-01-01) in the OBO format (ftp://ftp.geneontology.org/go/ontology-archive/). In addition, we downloaded time-matched Gene Ontology annotation data from UniProt-GOA (http://www.ebi.ac.uk/GOA; see the Frequently Asked Questions on http://www.geneontology.org for this and other options to access older versions of gene association files). Specifically, we downloaded UniProt-GOA version 116 as a time match for the 2012-12-01 ontology file, and UniProt-GOA version 164 (2017-01-16) for the 2017-01-01 ontology file. The Ross et al. dataset [26] was obtained from CilDB [27] and the resulting list of Ensembl protein identifiers were converted to gene symbols in Ensembl biomart (version 86) [28]. Term enrichment analysis was performed using the Ontologizer 2.1 [29] using the Parent–Child–Union method and applying the Bonferroni multiple testing correction. A custom R script was used to generate graphs to compare two term enrichment analyses for the same dataset with different combinations of GO and UniProt-GOA versions to investigate the effects of improvements in ontology and annotations separately and combined. The final graphs were processed in Adobe Illustrator for enhanced clarity. All scripts, required files, and instructions to obtain third party software are available on GitHub (https://github.com/JohnvanDam/GeneOntologySupplement).

## Results

### Improvements to cilia/flagellar Gene Ontology terms

As part of the SYSCILIA research Consortium [16], we examined the status of the cilia representation in GO at the end of 2012. Several discrepancies with current knowledge were highlighted, the main ones being as follows: (a) eukaryotic flagella were represented by the same concepts as prokaryotic flagella; (b) eukaryotic flagella were treated as separate from eukaryotic cilia; (c) two distinct terms existed for ‘cilium axoneme’ and ‘axoneme’, with the latter not being connected to the higher-order cilium structure; (d) the detailed substructure of the organelle, as well as basic cilia-related processes, were largely undocumented in GO, therefore limiting usefulness of the resource in many areas of basic research, but especially in the field of ciliopathies.

The issues above were addressed in collaboration with the Gene Ontology (GO) Consortium. As a result, many improvements were made to the ontology. The links between terms for eukaryotic flagellum and bacterial flagellum were removed, a term for archaeal flagellum was added, and we merged the eukaryotic flagellum and cilium terms into GO:0005929 ‘cilium’. Overall, 30 GO terms specifically related to prokaryotic flagella, and covering subcellular components as well as biological processes, are currently available in the Gene Ontology. They are listed in Additional file 2, and include 10 terms added or modified as part of this project. Previous annotations to cilium/flagellum terms were re-assigned where necessary based on taxonomy (i.e. bacterial, archaeal or eukaryotic).

**Fig. 1:**
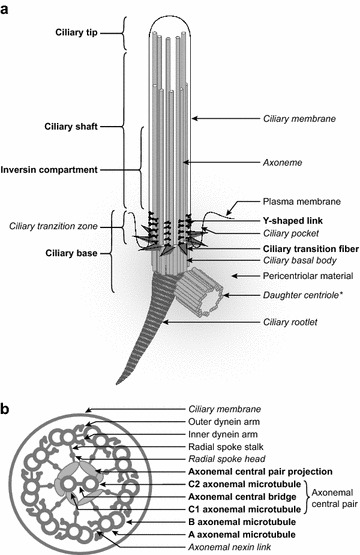
Schematic representation of the cilium and its main parts. Components in bold indicate new terms in GO; components in italics indicate pre-existing GO terms that were modified to improve them. **a** Schematic overview of a cilium. **b** Cross section of a cilium with a 9 + 2 axoneme. *‘Daughter centriole’ is a new synonym of ‘ciliary basal body’

**Fig. 2:**
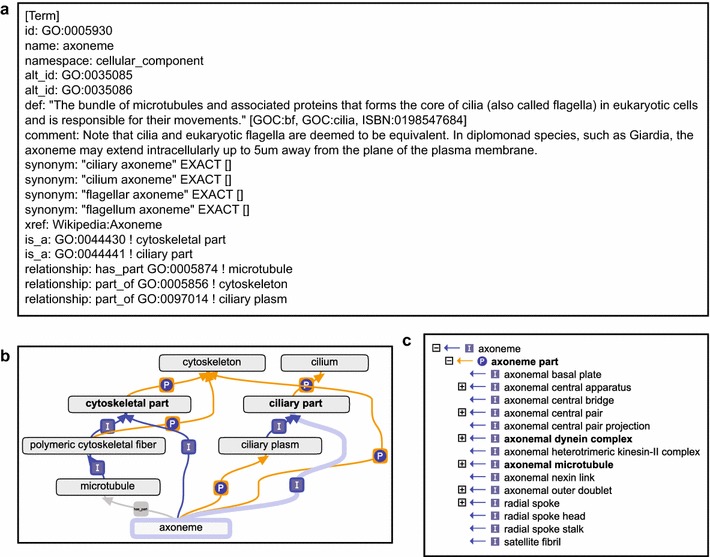
Details of the Gene Ontology term ‘axoneme’. **a** Full ontology stanza in OBO format. Documentation on relationship types and ontology format is available via [30]. **b** Placement of ‘axoneme’ within the Gene Ontology. The term itself and its link to ‘ciliary part’ are highlighted in light blue. Dark blue arrows and “I” indicate is_a relationships; orange arrows and “*p*” indicate part_of relationships. The grey arrow and rectangle connecting ‘axoneme’ and ‘microtubule’ indicate has_part relationship. **c** Overview of main axonemal substructures in GO. These are is_a children terms of ‘axoneme part’. Terms with a ‘+’ sign have children themselves. Terms in bold in **b**, **c** have computable definitions [31]. **b**, **c** were obtained with the Graph Editor function of the OBO-Edit ontology editing tool [20]

In Fig. 1, we provide a graphical representation of the cilium, and highlighting some of the ontology terms that were added or modified as part of this project. We captured up-to-date knowledge about well-defined structures by adding terms to represent the Y-shaped linkers in the transition zone, the central pair of microtubules in the 9 + 2 axoneme, transition fibres and many more (see Additional file 3). To address another major concern, the term ‘cilium axoneme’ was merged into ‘axoneme’, and ‘axoneme’ was made a part_of ‘cilium’ (via an intermediate link with the grouping term ‘ciliary part’). As a result, all axonemal substructures are now correctly placed in the ‘cilium’ branch of GO, and annotations to axonemal sub-components can now be propagated to ‘cilium’, with a positive impact on data analysis (e.g. enrichment studies). Figure 2 shows the Gene Ontology representation of GO:0005930 ‘axoneme’.

**Fig. 3:**
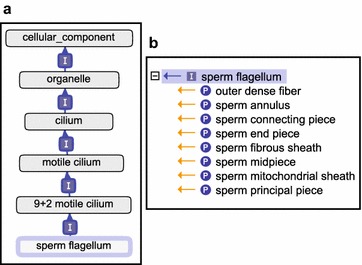
Details of the Gene Ontology term ‘sperm flagellum’. **a** Placement of ‘sperm flagellum’ within the Gene Ontology. The term itself and its link to the parent ‘9 + 2 motile cilium’ are highlighted in light blue. Dark blue arrows and “I” indicate is_a relationships. **b** Overview of main sperm flagellum substructures in GO (part_of children terms). Obtained with the Graph Editor function of the OBO-Edit ontology editing tool [20]. Documentation on relationship types is available via [30]

Similarly, we updated the representation of the well-studied mammalian sperm flagellum by placing it under a new, descriptive term ‘9 + 2 motile cilium’ (see below) and by adding missing connections to some of its sub-structural components; the improved hierarchy is shown in Fig. 3. We also implemented several ontology terms that occur in the literature and that do not refer to specific structures, but rather to observed ciliary subcompartments, such as the ‘inversin compartment’ [32], ‘ciliary tip’ [33] and ‘ciliary base’ [34]. In Additional file 3, we provide a full list of GO terms currently available to the scientific community to describe ciliary subcompartments and main cilia-related biological processes, for a grand total of 180 classes as of January 2017. Of these, 65% (117 terms) were created or modified as part of the ontology development project described here. While the curation of human ciliary proteins using GO terms is described below, it is worth noting here that 54% of all existing cilia-related GO terms applicable for mammalian annotation have been used to annotate mouse proteins in a parallel complementary effort (Christie and Blake [17]).

We examined how cilia types were categorized in GO, and revised and expanded that classification significantly. Previously, GO:0005929 ‘cilium’ had two children, ‘motile cilium’ and ‘primary cilium’, with descendants ‘motile primary cilium’ and ‘nonmotile primary cilium’. That categorization was thus trying to capture both motility and sensory aspects of cilia at the same time. However, in doing so, it did not allow for a complete and correct representation of current knowledge. For example, specialized cilia in vertebrate embryos, e.g. nodal cilia of the mouse or cilia in the Kupffer’s vesicle of zebrafish, are motile, but have an axoneme configuration of 9 + 0, often found in non-motile cilia [35]; conversely, kinocilia display a 9 + 2 axonemal structure, but are considered non-motile [4]. Also, motile cilia have been shown to have a variety of sensory functions [36].

**Fig. 4:**
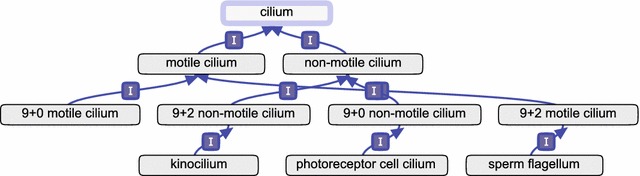
Details of the Gene Ontology term ‘cilium’ and its is_a descendants. The term ‘cilium’ itself is highlighted in light blue. Dark blue arrows and “I” indicate is_a relationships. Obtained with the Graph Editor function of the OBO-Edit ontology editing tool [20]

We reviewed the literature, and resolved to classify cilia based primarily on the presence or absence of motility, and secondarily on their axonemal configuration. The role of cilia in sensory pathways, when present, should instead be captured by annotating to appropriate biological process terms, rather than trying to embed it in a cellular component term. The classification we implemented is consistent with the recent one by Takeda and Narita, who proposed an eight-category system based on axonemal configuration, motility of the cilium, and number of cilia per cell [37]. For the Cellular Component branch of GO, only the structural aspects of axonemal configuration and motility are relevant, so we simplified to a four-category system. A similar four-category classification was also proposed by Ibañez-Tallon et al. [38] and supported by Fisch and Dupuis-Williams [39]. We also consulted directly with some experts in the cilia community, and presented our proposal at the international Cilia 2016 conference held in Amsterdam, The Netherlands [40]. Fig 4 shows the current ontology structure. Note that the GO classification does not aim to include individual terms for the totality of axonemal configurations observed in nature (such as 9 + 4 axonemes in Hensen’s node in rabbit embryos [41], or some unusual structures observed in insects [42]), but still allows the capture of less common instances as specifically as possible, as well as ones where fine structure or motility are not known.

**Fig. 5:**
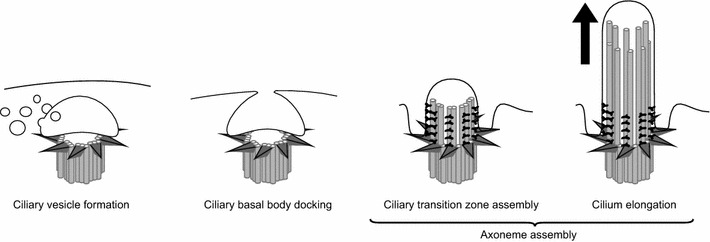
Cilium assembly. In vertebrates, the ciliary vesicle forms at the tips of the ciliary transition fibres attached to the basal body. The ciliary vesicle then fuses with the plasma membrane forming the ciliary pocket and ciliary membrane. The axoneme extends from the basal body and the transition zone is assembled with its distinctive Y-shaped links and ciliary necklace. Further axonemal assembly causes the cilium to elongate

Due to the increasing number of cellular pathways in which cilia are known to be involved, the Biological Process branch of GO was also in need of improvement. We focussed mainly on two distinct areas: cilium organization and multiciliation. Within the first area, we revised the ontology under the ciliogenesis branch (GO:1903887 ‘cilium assembly’) by aligning it with the manually curated Reactome Pathway Database. Reactome entries are authored by expert biologists in collaboration with Reactome editorial staff, and cross-reference to many bioinformatics databases [43, 44]. Therefore, structuring GO processes in agreement with Reactome (and vice versa) increases interoperability and optimizes the engagement of field researchers, while still maintaining specific scopes for each resource (in GO, representation of pathways focuses on processes encoded by gene products, whereas in Reactome it is centred on transformations of chemical entities). The Reactome entry for ‘Assembly of the primary cilium’ was revised recently, and captures up-to-date knowledge [45]. We worked with Reactome editors to improve integration with GO in this area; for example, Reactome renamed their entry to ‘Cilium Assembly’ to reflect applicability to cilium subtypes in agreement with GO classification. New GO terms were created as necessary, and links among GO terms were added, resulting in a richer representation of the biological events that lead to the formation of a cilium. GO terms that had corresponding Reactome entries were cross-referenced with appropriate Reactome identifiers, and vice versa. (Due to the different natures of these resources, not all terms can be linked effectively.) We also extended the cilium assembly ontology representation by including the formation of the intermediate ciliary vesicle as observed in vertebrates [46] (Fig. 5). GO terms available to describe details of the cilium assembly process are included in Additional file 3.

The revision of the overall ‘cilium organization’ process branch of GO (GO:0044782) impacted an existing term, ‘cilium morphogenesis’. We found that, in view of the new, more detailed representation of ciliary processes in GO, the meaning of ‘cilium morphogenesis’ now referred to a mixture of ‘cilium assembly’ and its parent term ‘cilium organization’. We removed the now-redundant class ‘cilium morphogenesis’, and worked with GO biocurators to rehouse its previous annotations (to several different species) under the most appropriate terms.

Among cilia-related processes, we also focused on those that lead to formation of multiciliated cells. Following discussions with members of the cilia research community, it became clear that the distinction between uniciliated and multiciliated cells was biologically important. However, this feature could not be incorporated as such in the Cellular Component branch of GO, as the cilia in multiciliated cells are generally not structurally distinct from those in singly ciliated cells. Rather, ‘multiciliation’ is a complex and multifaceted cell differentiation process that occurs in specific tissues or organisms, and that was previously only minimally represented in GO. We improved its description in several ways, for example by adding to the branch of ‘de novo centriole assembly’ (see Additional file 3). It is also important to note that, when capturing the role of multiciliation proteins via GO annotation, curators can increase expressivity of their annotations, wherever possible, to indicate the specific cell type(s) in which the protein functions. This is accomplished by referring to the Cell Ontology [47], which provides a broad coverage of ciliated cell classes, and using a compositional approach described by Huntley et al. [24].

Another area that received attention was ‘cilium-dependent cell motility’ (GO:0060285). Terms related to bacterial, archaeal, and eukaryotic flagellar/ciliary-based cell motility were made distinct from each other. We carried out a revision to better describe the mechanism of mobility, including cases that do not involve flagellated cells, such as ‘amoeboid sperm motility’ (observed in e.g. the sperm of C. elegans [48, 49]). Overall, 5 new terms were added to account for instances of non-ciliated sperm motility (generic ‘sperm motility’, ‘amoeboid sperm motility’, and regulation terms for the latter); these are not included in the list of cilia-related terms available in Additional file 3.

Overall, as part of the work described in this paper, we added 76 new ontology terms related to cilia or flagella, and modified 51 existing ones. Additional file 3 provides the complete list of cilia- and flagella-related Cellular Component and Biological Process terms that are now available for data analysis and to capture ciliary and flagellar biology. Full details of ontology terms (including synonyms and relationships with other terms) are publicly accessible via the GO browsers AmiGO and QuickGO [50, 51]. The ontology may be downloaded freely from http://geneontology.org/page/download-ontology.

### Concurrent gene annotation efforts

In order for the improved ontology to have an impact, genes and gene products need to be annotated using these new terms. Using the ontology for annotation also helps to clarify what terms are needed in the ontology. For our annotation effort, we started with a set of twelve genes from the SCGS involved in ciliary movement, primarily dyneins and genes involved in axoneme assembly [15] (DNAH1, DNAH11, DNAH5, DNAH9, DNAI1, DNAI2, CCDC114, CCDC39, CCDC40, DISC1, NME8, and PCM1; UniProt identifiers Q9P2D7, Q96DT5, Q8TE73, Q9NYC9, Q9UI46, Q9GZS0, Q96M63, Q9UFE4, Q4G0X9, Q9NRI5, Q8N427 and Q15154, respectively). Our literature searches identified 27 relevant papers for these genes, as well as two additional papers focused on two genes (ARMC4 and DNAH7, with UniProt IDs Q5T2S8 and Q8WXX0) that are also associated with primary ciliary dyskinesia. From these 29 papers (Additional file 4; also see below), we made 157 annotations, 89 of which were to ciliary GO terms for 40 human genes (Additional files 5, 6; also see below). A few of these papers also included experimental characterization of mouse genes; annotations made for mouse genes are included in the annotation project described by Christie and Blake [17].

In the process of making phylogenetic annotations, as described below, we identified proteins in *Chlamydomonas reinhardtii* that had been experimentally studied and could be used to infer functions for uncharacterized homologs in humans and other animals. Most of these proteins are inner arm or outer arm axonemal dyneins or the cytoplasmic type dyneins involved in intraflagellar transport (IFT). Thus, we annotated 13 papers (Additional file 7) with experimental characterizations of ciliary dyneins from *Chlamydomonas reinhardtii*. This produced 74 annotations (55 to ciliary terms) to 16 dynein genes, as well as 3 other genes (Additional files 8, 9). We also annotated four additional papers (Additional file 4) targeting the human genes DYNC2H1 and WDR60 (UniProt IDs Q14204 and Q8WVS4). This follow-up work making literature-based annotations generated 42 more annotations to 10 additional human genes, bringing our total to 199 GO annotations (Additional file 6) for 50 human genes (Additional file 5).

Concurrent to our efforts, Christie and Blake have fully curated 134 mouse ciliary genes, all of which correspond to human genes on the SCGS list, as of December 2016 (Christie and Blake [17]). Amongst the genes targeted for annotation in that project were the majority of the dynein genes on the list of mouse homologs of SCGS human genes, focusing on those not previously well annotated. While many of the GO annotations for these genes were to processes that are affected when cilia are disrupted, such as ‘determination of left/right symmetry’ or ‘cilium movement’, some were to terms useful for the phylogenetic annotation of dynein proteins.

This solid base of experimental annotations for human and *Chlamydomonas* dynein genes, as well as a few from mouse, allowed us to make detailed phylogenetic annotations using the Phylogenetic Annotation and INference Tool [52] of the sequences within the seven PANTHER protein families [9] containing ciliary dynein genes (Additional file 10). A couple of the smaller dynein families had previously been annotated, but our additional annotations allowed propagation of GO terms providing specificity with respect to which type of dynein complex(es) are relevant. However, the majority of the dynein sequences, including those in the large families for dynein heavy chains (PTHR10676), dynein intermediate chains (PTHR12442), or dynein light chains (PTHR11886), had not previously been phylogenetically annotated. Thus, our annotations provided the basis for comprehensive phylogenetic annotation of the ciliary dynein genes. Up-to-date GO annotations may be freely downloaded from the GO website [53] or using QuickGO [51].

### Effects of Gene Ontology and protein annotation improvements on term enrichment analyses

In order to assess the effects of our improvements on the practical utility of the GO resource for ciliary researchers, we performed GO term enrichment analysis on two published datasets using versions of GO ontology and annotations from December 2012, when we started the project, and January 2017, and comparing the results. We used the software package Ontologizer [29] to perform GO term enrichment analyses using the corresponding sets of Gene Ontology annotations from UniProt [25]. Two datasets were considered: the SYSCILIA Gold Standard of cilia genes [15], and a gene expression dataset of reassembling motile cilia in lung epithelial cells by Ross et al. [26].

**Fig. 6:**
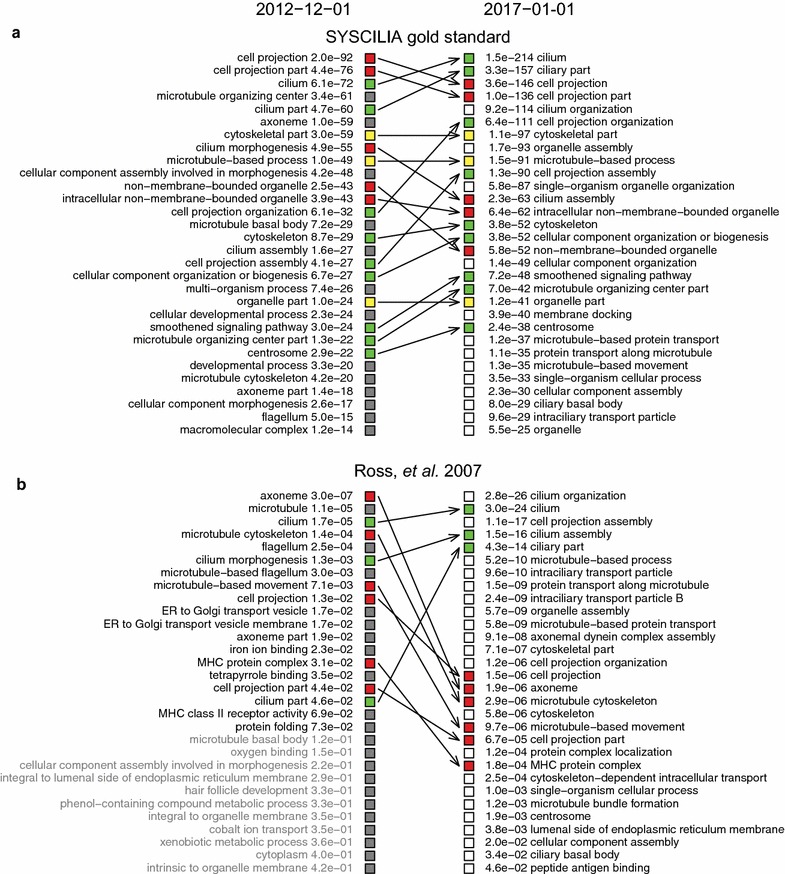
Comparison of GO term enrichment analyses of ciliary datasets using versions of GO from 2012 and 2017. Green squares: GO terms that rank higher using the current version of GO; red squares: terms that rank lower; grey squares: terms that have dropped out of the top 30 ranked results; white squares: terms that are among the top 30 when using the current version of GO, but not the 2012 one. *p* values were corrected using the Bonferroni multiple testing correction. Terms in grey are not significantly enriched. **a** Term enrichment analyses of the SYSCILIA gold standard. Cilia-specific terms rank higher. The improvement of the Gene Ontology and advancement in gene annotations have also been assessed respective of each other, see Additional file 11. **b** Term enrichment analyses of the Ross et al. dataset. Overrepresented terms gained smaller *p* values but have also become more descriptive of the experiments, e.g. ‘cilium organization’, ‘cellular component assembly involved in morphogenesis’ and ‘cilium assembly’

The SCGS is a standardized list of verified ciliary genes for use in systems biology approaches [15]. The improvements in ontology are reflected in two ways in a GO term enrichment analysis for this dataset (Fig. 6a). Terms directly related to the cilium appear consistently higher in the ranking. Using the current state of GO ontology and annotations, ‘cilium’ is now the top ranking term. Equally important is the observed lower *p* value (6.1e−72 in December 2012 vs. 1.5e−214 in January 2017). A significant contribution to the improvement of observed *p* values is brought by the concurrent mouse annotation effort by Christie and Blake [17], in which the list of genes targeted for annotation was based on the SCGS. Mouse annotations were subsequently transferred to their human 1-to-1 orthologs and assigned an evidence code ‘Inferred from Sequence Orthology’ (ISO), according to an established pipeline described in [54]. The ontology development and annotation work described in this paper, and the mouse annotation project carried out by Christie and Blake, act synergistically towards a better representation of up-to-date knowledge of the cilium. To illustrate the respective contribution of the progress in GO annotation and ontology development, we performed GO term enrichment analysis using the current ontology but the old 2012 gene annotations, and then using the current annotations but the old ontology version from 2012 (see Additional file 11). These analyses clearly show the significant impact of the progress in both gene annotation and ontology development, on the ranking as well as the *p* values of relevant ciliary terms.

In our second analysis, Ross et al. describe a gene expression study of human airway epithelial cells cultured at an air–liquid interface [26]. The culture conditions cause differentiation into multiciliated cells; thus, the gene expression dataset is expected to reflect the molecular processes involved in cilium assembly, the process of forming cilia. In the 2012 state of GO ontology and annotations, ciliary-related terms are already significantly overrepresented (Fig. 6b). However, using the current version of GO, we find more relevant GO terms descriptive of the processes that the experiments were designed to examine, such as ‘cilium organization’ and ‘cilium assembly’ (Fig. 6b). Overall, the overrepresented ciliary terms have not only become higher in ranking with smaller *p* values, but also more specific.

## Discussion

The importance of cilia in a vast array of cell types across eukaryotes, and their role in an ever-growing number of human diseases and disorders, prompted us to address the gap between current knowledge on ciliary structures and processes and the Gene Ontology (GO), the most widely used tool to represent this knowledge computationally and make it available to the biomedical research community. Our effort increased the number of ontology classes available to describe cilia, flagella and the events they participate in, and allowed a significant improvement in the curation coverage of mammalian ciliary factors.

Our project enables a more consistent representation of knowledge by providing the community with an ontology structure that includes a standardized set of concepts that are carefully defined and related to each other. In fact, while term usage in the scientific literature can sometimes be ambiguous, GO requires its classes to be unambiguously defined. An example is the frequent use of “axonemal localization” in articles, meaning “localization along the length of the cilium”. However, “axonemal localization” could also be interpreted such that a protein specifically is “part of” the ciliary axonemal microtubule structures. The former interpretation of the term may be clear to scientists comfortable with cilia research, but not to those new to or outside of the field. The formalization in GO must be accessible to a broad scientific community, and in this case includes several terms to denote specific regions of the cilium. For instance, we defined the sporadically used term ‘ciliary shaft’ to correspond to the protruding part of the cilium, and thus this term is often a better representation of what is meant when a protein is observed to “localize to the axoneme”.

Some of the new GO terms we implemented will make it easier to represent experimental findings from the literature when resolution issues prevent assignment to well-defined ciliary compartments. For example, GO now provides the term ‘ciliary base’ that denotes a more general location when the experimental (e.g. microscopy) observations are not precise enough to define protein localization to more specific ciliary compartments such as the basal body, transition fibres or transition zone.

Importantly, the ontology development we carried out also improved connections among existing classes. This has a positive downstream effect on data analysis. For example, by connecting ‘axoneme’ to ‘cilium’ via the part_of relationship, pre-existing GO annotations to the former are automatically inferred to the latter, improving the sensitivity of enrichment analyses. Similarly, merging terms that represent the same entity (such as ‘cilium axoneme’ and ‘axoneme’) solved the issue of fragmentation of GO annotations over multiple terms. This, too, positively impacts data analysis.

There is always the potential to add more terms as new knowledge emerges or where more precise representation of existing knowledge is requested by the community. For example, species-specific axonemal arrangements that are not currently present in GO (e.g. 9 + 4 axonemes in Hensen’s node in rabbit embryos [41]) could be incorporated if deemed useful to support data analysis.

The improved GO vocabulary is being actively used to describe experimental findings for human and mouse ciliary proteins, consistent with the focus of the GO Consortium on representing human biology. In this way, ciliary genes and gene products are now being integrated into gene and protein networks to provide productive insight into biomedical studies where cilia and flagella are involved. Some of the GO terms we created or modified have already been used to annotate human genes in the SYSCILIA Gold Standard set.

Terms of the improved GO vocabulary have also been used extensively to annotate ciliary proteins of the mouse, one of the best systems for generation of models for human genetic diseases [55, 56]. For example, the many publications describing research into mouse models of retinal degeneration provided impetus to improve the representation of the photoreceptor cilium, including the knowledge that the ‘photoreceptor connecting cilium’ is a specialized type of ‘ciliary transition zone’. These improvements greatly increased our ability to capture experimental work characterizing mouse models that advances the understanding of a devastating human disease. In this manner, annotation of mouse genes fed back into the development of the ontology, both to clarify previously existing terms or to create new terms when needed (Christie and Blake [17]).

Many of the ontology revisions we made also improve information available for other species, and further improvements can be made as the need arises. Notably, for protein families where experimental characterization is lacking in human and mouse (such as some dyneins), we curated experimental information available from a non-mammalian organism (*Chlamydomonas reinhardtii*). These experimental annotations also enabled the phylogenetic inference of GO annotations via a dedicated and validated pipeline, both to species of biomedical interest and also to many more species where direct characterization of ciliary proteins is unlikely. We also worked to reflect the fact that cilia have not been observed in some taxonomic groups, e.g. some types of plants (including Magnoliophyta, Coniferophyta, and Gnetales), slime molds (*Dictyostelium*), and most fungi (including Ascomyceta). In such cases, we applied computational rules to prevent usage of some general ciliary terms (e.g. ‘cilium’, ‘cilium assembly’ and ‘cilium movement’) for annotation in non-ciliated species. The presence of these taxonomic rules helps ensure correctness of annotations [57], as checks can be applied both during manual annotation of experimental literature and during phylogenetic annotation pipelines.

Another way that our work improved the information available for other species was in areas of the ontology where we uncovered flaws in the original scope of GO terms or the structure relating GO terms to each other, such that the addition of new terms was required in order to provide clarity. One such area was that of flagella generally, where the previous ontology structure had conflated bacterial flagella with those of eukaryotes, and also made an inappropriate distinction between eukaryotic cilia versus eukaryotic flagella. The resolution of this problem generated new terms or clarified existing ones specifically for the use in annotation of either bacterial or archaeal species, as appropriate. In addition, the term ‘cilium or flagellum-dependent cell motility’, a grouping term for cell motility via any type of cilia or flagella, was marked with a tag indicating that it is inappropriate for manual annotation as eukaryotic cilia and bacterial flagella never co-exist in the same organism; thus it should always be possible for the biocurator to select the appropriate more specific term based on which type of organism is being annotated.

We uncovered another logical flaw in the ontology while trying to make a connection between ‘sperm motility’ and ‘cilium-dependent cell motility’. We realized that there is more than one mechanism of sperm motility, either flagellated or amoeboid (note that the non-flagellated sperm present in many plant species are not themselves motile cells and are instead moved by the pollen tube). Thus, our addition of GO terms to describe amoeboid sperm motility will be useful to properly annotate gene products involved in the motility of amoeboid sperm in nematodes such as *C. elegans*.

## Conclusions

The enhanced ciliary ontology, and the improvements in breadth and depth of gene annotation, will allow more precise knowledge representation, which in turn will generate more informative results from data analyses. The latter can potentially include re-analysis of existing datasets, maximizing the usefulness of experimental work for the scientific community and ultimately leading to significant advances in our understanding of biology. This is especially important considering the increasing focus on ciliopathies, as apparent from the steady yearly increase in the number of publications on the subject since 2006 (see Additional file 1). The advantages of applying similar focused curation approaches to cell organelles were also shown recently for the peroxisome [58].

Our work lays solid foundations for the usefulness of GO (and GO annotations) as a powerful resource for ciliary researchers. In fact, beyond the informative classes to describe cilia structure and processes such as cilium assembly, which were the object of this project, the GO also represents other processes relevant to this organelle. A partial list includes signalling pathways, developmental processes and sensory perception events involving cilia. In fact, due to the numerous roles the cilium plays in many developmental and signalling pathways, many processes involving ciliary function may still benefit from improvement of ontology and annotation. Also, because the effort described here focused mostly on mammals, there is still space in GO to expand representation of ciliary structures found in other species. Input from research experts on these individual processes will be needed, since they possess the specialized knowledge to help guide ontology development to reflect the biology accurately. Research communities within the ciliary field are invited to collaborate in joint projects with the GO consortium to tackle specific areas of GO related to cilia. The GO consortium also welcomes individual contributions by external experts (see http://geneontology.org/page/contributing-go).

## Additional files

**Additional file 1.** Number of publications on ciliopathies as recorded in PubMed using the search term ‘ciliopath*’ (to include ciliopathy, ciliopathies, etc.). Points represent available data (incomplete data for 2017 is represented by a dark grey point). A local polynomial regression fit of the publication data allows for predicting the number of publications for 2017 (black line; standard errors of the fit are represented as a grey ribbon).

**Additional file 2.** Prokaryotic flagella-related cellular component and biological process terms in the Gene Ontology (GO). Full list of terms available in the Gene Ontology (GO) to describe cellular components and biological processes related to prokaryotic flagella and available as of January 2017. Terms are listed in 3 separate sections pertinent to cellular components, motility and general organization of the flagellum, respectively. GO ID, Gene Ontology term identifier; GO term name, Gene Ontology term descriptive label; Aspect, branch of the Gene Ontology (CC = cellular component, BP = biological process); Created/modified by cilia GO project, indicates if a term was created or modified as part of the work described in this article. All GO terms can be browsed via AmiGO (http://amigo.geneontology.org/amigo) or QuickGO (http://www.ebi.ac.uk/QuickGO/), and the full ontology can be downloaded via http://geneontology.org/page/download-ontology.

**Additional file 3.** Cilia-related cellular component and biological process terms in the Gene Ontology (GO). Full list of terms available in the Gene Ontology (GO) to describe cilia-related cellular components and biological processes as of January 2017, ordered alphabetically by term name. GO ID, Gene Ontology term identifier; GO term name, Gene Ontology term descriptive label; Created/modified by cilia GO project, indicates if a term was created or modified as part of the work described in this article. All GO terms can be browsed via AmiGO (http://amigo.geneontology.org/amigo) or QuickGO (http://www.ebi.ac.uk/QuickGO/), and the full ontology can be downloaded via http://geneontology.org/page/download-ontology.

**Additional file 4.** Papers used to make annotations for human ciliary genes. Full list of papers with corresponding PubMed IDs that were used for annotations for human ciliary genes. The first 29 papers comprise the set initially selected for the annotation project. The last four were curated later and specifically targeted to fill in gaps in experimental annotations of certain orthology groups in order to be able to propagate highly specific dynein-related annotations to other sequences within those orthology groups.

**Additional file 5.** Human genes annotated. List of all human genes annotated during this project, ordered alphabetically by Gene Symbol, with UniProtKB ID and Gene Name information, and presence within the SYSCILIA Gold Standard (SCGS) set also included. The numbers of experimental annotations made by this work to either cilia subset terms or to other GO terms are indicated. The Comment field indicates which genes were targeted in the initial set of papers, which were targeted for supplemental annotations, and also which genes are candidates to add to the SCGS based on the experimental work annotated by this project.

**Additional file 6.** Gene Association File for Human Annotations. File in GAF 2.0 format (http://www.geneontology.org/page/go-annotation-file-format-20) containing all the human annotations made by this project. Annotations for the 33 publications listed in Additional file 3 were extracted by grepping for the relevant PMID’s from the goa_human.gaf file generated on 3/13/2017 as downloaded from http://www.geneontology.org/page/download-annotations on 3/20/2017. A subsequent step to filter the annotations by source (column 15) to either SYSCILIA_CCNET or GO_Central generated this file of annotations generated for human genes for this project.

**Additional file 7.** Papers used to make annotations for *Chlamydomonas reinhardtii* ciliary genes. Full list of papers with corresponding PubMed IDs that were used for annotations for *Chlamydomonas reinhardtii* ciliary genes.

**Additional file 8.** *Chlamydomonas reinhardtii* genes annotated. List of all *Chlamydomonas reinhardtii* genes annotated during this project, with the UniProtKB ID, status of the UniProt record, Gene Symbol (if available), and Protein Name information. The numbers of experimental annotations made by this work to either cilia subset terms or to other GO terms are indicated. The Comment field indicates the experimentally demonstrated location in the axoneme as captured by the experimental annotations generated by this project.

**Additional file 9.** Gene Association File for *Chlamydomonas reinhardtii* annotations. File in GAF 2.0 format (http://www.geneontology.org/page/go-annotation-file-format-20) containing all the *Chlamydomonas reinhardtii* annotations made by this project. These annotations were extracted from the goa_uniprot_all_noiea.gaf file generated on 2/13/2017 as downloaded from http://www.geneontology.org/page/download-annotations on 3/17/2017. Note that subsequent to this being generated, all annotations for A8JDH8 were transferred to Q4AC22, a more complete sequence for DHC9 and all annotations for A8JDN3 were transferred to Q39604, the SwissProt reviewed entry for IDA4. Additionally, in response to our suggestion, A8JDN3 was merged into Q39604 as it was identical in sequence.

**Additional file 10.** Panther protein families. List of Panther protein families (version 11.1) containing dynein subunit sequences, included Panther family ID and family name, the total number of sequences in each family, the human and *Chlamydomonas* genes present in each. We have indicated the annotation date as well as the impact of our experimental annotations on the phylogenetic annotations propagated by the PAINT curation process.

**Additional file 11.** Additional GO term enrichment analysis illustrating the effect of improved GO ontology and annotations separately. Six GO enrichment comparisons are shown of which two are also depicted in Fig. 6. In the four additional comparisons, the 2012 state of GO ontology and annotations is compared to either the current ontology using old annotations from 2012, or the ontology from 2012 but with the current annotations. The former comparison illustrates the effects of the improved GO terminology and the underlying connections of terms, while the latter comparison illustrates the effects of increased gene annotations over the same period of time. *p* values are corrected using the Bonferroni multiple testing correction. Terms in grey are not significantly enriched.

## Abbreviations

BP: biological process
CC: cellular component
EMBL-EBI: European Molecular Biology Laboratory, European Bioinformatics Institute
GO: Gene Ontology
GOC: Gene Ontology Consortium
MF: molecular function
OBO: open biomedical ontologies
PAINT: Phylogenetic Annotation and INference Tool
PANTHER: Protein ANalysis THrough Evolutionary Relationships
SCGS: SYSCILIA Gold Standard
SYSCILIA: a systems biology approach to dissect cilia function and its disruption in human genetic disease
UniProt-GOA: Gene Ontology Annotation Database at the Universal Protein Resource
