# Supplementary figures and images for: The Gene Ontology of eukaryotic cilia and flagella
Source: Cilia. 2017 Nov 16;6:10. doi: 10.1186/s13630-017-0054-8 (PMC5688719; doi:10.1186/s13630-017-0054-8)

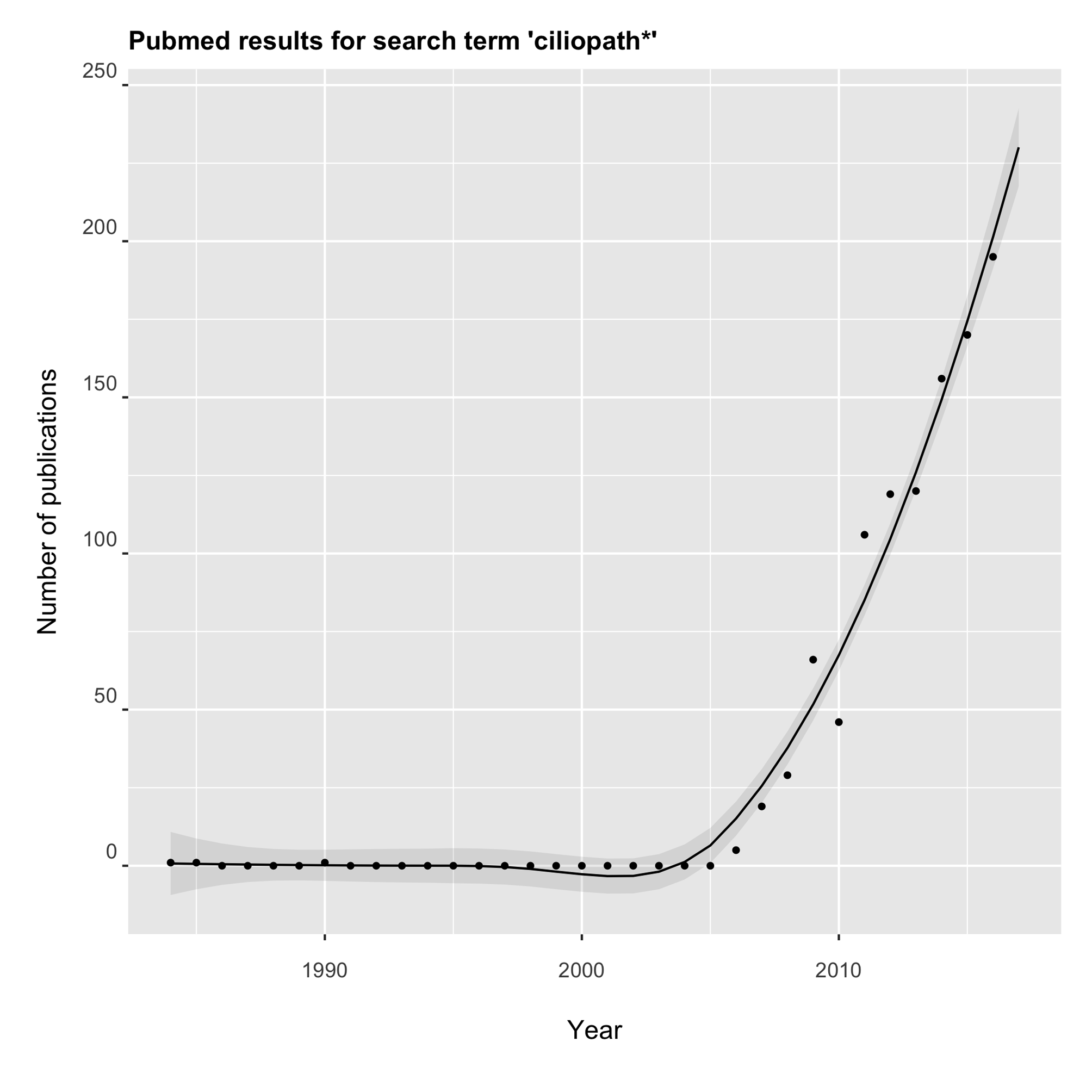

Supplement: Supplementary file 1 — Additional file 1. Number of publications on ciliopathies as recorded in PubMed using the search term ‘ciliopath*’ (to include ciliopathy, ciliopathies, etc.). Points represent available data (incomplete data for 2017 is represented by a dark grey point). A local polynomial regression fit of the publication data allows for predicting the number of publications for 2017 (black line; standard errors of the fit are represented as a grey ribbon). [file 13630_2017_54_MOESM1_ESM.png]
