# Supplementary material for: The Gene Ontology of eukaryotic cilia and flagella
Source: Cilia. 2017 Nov 16;6:10. doi: 10.1186/s13630-017-0054-8 (PMC5688719; doi:10.1186/s13630-017-0054-8)
Supplement: Supplementary file 11 — Additional file 11. Additional GO term enrichment analysis illustrating the effect of improved GO ontology and annotations separately. Six GO enrichment comparisons are shown of which two are also depicted in Fig. 6. In the four additional comparisons, the 2012 state of GO ontology and annotations is compared to either the current ontology using old annotations from 2012, or the ontology from 2012 but with the current annotations. The former comparison illustrates the effects of the improved GO terminology and the underlying connections of terms, while the latter comparison illustrates the effects of increased gene annotations over the same period of time. p values are corrected using the Bonferroni multiple testing correction. Terms in grey are not significantly enriched. [file 13630_2017_54_MOESM11_ESM.pdf]

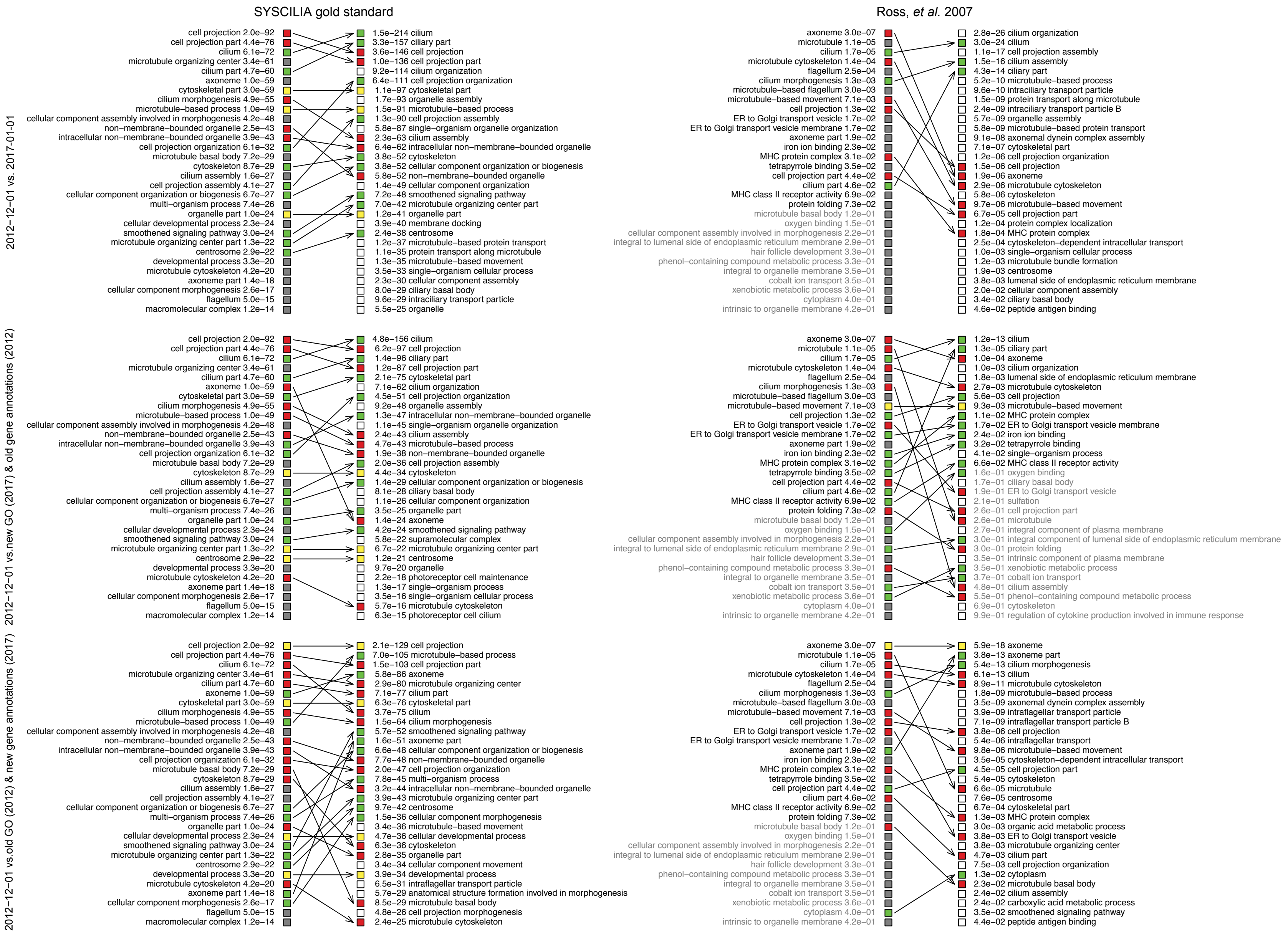

SYSCILIA gold standard
Ross, et al. 2007
2012−12−01 vs. 2017−01−01
cell projection 2.0e−92
cell projection part 4.4e−76
cilium 6.1e−72
microtubule organizing center 3.4e−61
cilium part 4.7e−60
axoneme 1.0e−59
cytoskeletal part 3.0e−59
cilium morphogenesis 4.9e−55
microtubule−based process 1.0e−49
cellular component assembly involved in morphogenesis 4.2e−48
non−membrane−bounded organelle 2.5e−43
intracellular non−membrane−bounded organelle 3.9e−43
cell projection organization 6.1e−32
microtubule basal body 7.2e−29
cytoskeleton 8.7e−29
cilium assembly 1.6e−27
cell projection assembly 4.1e−27
cellular component organization or biogenesis 6.7e−27
multi−organism process 7.4e−26
organelle part 1.0e−24
cellular developmental process 2.3e−24
smoothened signaling pathway 3.0e−24
microtubule organizing center part 1.3e−22
centrosome 2.9e−22
developmental process 3.3e−20
microtubule cytoskeleton 4.2e−20
axoneme part 1.4e−18
cellular component morphogenesis 2.6e−17
flagellum 5.0e−15
macromolecular complex 1.2e−14
1.5e−214 cilium
3.3e−157 ciliary part
3.6e−146 cell projection
1.0e−136 cell projection part
9.2e−114 cilium organization
6.4e−111 cell projection organization
1.1e−97 cytoskeletal part
1.7e−93 organelle assembly
1.5e−91 microtubule−based process
1.3e−90 cell projection assembly
5.8e−87 single−organism organelle organization
2.3e−63 cilium assembly
6.4e−62 intracellular non−membrane−bounded organelle
3.8e−52 cytoskeleton
3.8e−52 cellular component organization or biogenesis
5.8e−52 non−membrane−bounded organelle
1.4e−49 cellular component organization
7.2e−48 smoothened signaling pathway
7.0e−42 microtubule organizing center part
1.2e−41 organelle part
3.9e−40 membrane docking
2.4e−38 centrosome
1.2e−37 microtubule−based protein transport
1.1e−35 protein transport along microtubule
1.3e−35 microtubule−based movement
3.5e−33 single−organism cellular process
2.3e−30 cellular component assembly
8.0e−29 ciliary basal body
9.6e−29 intraciliary transport particle
5.5e−25 organelle
axoneme 3.0e−07
microtubule 1.1e−05
cilium 1.7e−05
microtubule cytoskeleton 1.4e−04
flagellum 2.5e−04
cilium morphogenesis 1.3e−03
microtubule−based flagellum 3.0e−03
microtubule−based movement 7.1e−03
cell projection 1.3e−02
ER to Golgi transport vesicle 1.7e−02
ER to Golgi transport vesicle membrane 1.7e−02
axoneme part 1.9e−02
iron ion binding 2.3e−02
MHC protein complex 3.1e−02
tetrapyrrole binding 3.5e−02
cell projection part 4.4e−02
cilium part 4.6e−02
MHC class II receptor activity 6.9e−02
protein folding 7.3e−02
microtubule basal body 1.2e−01
oxygen binding 1.5e−01
cellular component assembly involved in morphogenesis 2.2e−01
integral to lumenal side of endoplasmic reticulum membrane 2.9e−01
hair follicle development 3.3e−01
phenol−containing compound metabolic process 3.3e−01
integral to organelle membrane 3.5e−01
cobalt ion transport 3.5e−01
xenobiotic metabolic process 3.6e−01
cytoplasm 4.0e−01
intrinsic to organelle membrane 4.2e−01
2.8e−26 cilium organization
3.0e−24 cilium
1.1e−17 cell projection assembly
1.5e−16 cilium assembly
4.3e−14 ciliary part
5.2e−10 microtubule−based process
9.6e−10 intraciliary transport particle
1.5e−09 protein transport along microtubule
2.4e−09 intraciliary transport particle B
5.7e−09 organelle assembly
5.8e−09 microtubule−based protein transport
9.1e−08 axonemal dynein complex assembly
7.1e−07 cytoskeletal part
1.2e−06 cell projection organization
1.5e−06 cell projection
1.9e−06 axoneme
2.9e−06 microtubule cytoskeleton
5.8e−06 cytoskeleton
9.7e−06 microtubule−based movement
6.7e−05 cell projection part
1.2e−04 protein complex localization
1.8e−04 MHC protein complex
2.5e−04 cytoskeleton−dependent intracellular transport
1.0e−03 single−organism cellular process
1.2e−03 microtubule bundle formation
1.9e−03 centrosome
3.8e−03 integral to lumenal side of endoplasmic reticulum membrane
2.0e−02 cellular component assembly
3.4e−02 ciliary basal body
4.6e−02 peptide antigen binding
2012−12−01 vs.new GO (2017) & old gene annotations (2012)
cell projection 2.0e−92
cell projection part 4.4e−76
cilium 6.1e−72
microtubule organizing center 3.4e−61
cilium part 4.7e−60
axoneme 1.0e−59
cytoskeletal part 3.0e−59
cilium morphogenesis 4.9e−55
microtubule−based process 1.0e−49
cellular component assembly involved in morphogenesis 4.2e−48
non−membrane−bounded organelle 2.5e−43
intracellular non−membrane−bounded organelle 3.9e−43
cell projection organization 6.1e−32
microtubule basal body 7.2e−29
cytoskeleton 8.7e−29
cilium assembly 1.6e−27
cell projection assembly 4.1e−27
cellular component organization or biogenesis 6.7e−27
multi−organism process 7.4e−26
organelle part 1.0e−24
cellular developmental process 2.3e−24
smoothened signaling pathway 3.0e−24
microtubule organizing center part 1.3e−22
centrosome 2.9e−22
developmental process 3.3e−20
microtubule cytoskeleton 4.2e−20
axoneme part 1.4e−18
cellular component morphogenesis 2.6e−17
flagellum 5.0e−15
macromolecular complex 1.2e−14
4.8e−156 cilium
6.2e−97 cell projection
1.4e−96 ciliary part
1.2e−87 cell projection part
2.1e−75 cytoskeletal part
7.1e−62 cilium organization
4.5e−51 cell projection organization
9.2e−48 organelle assembly
1.3e−47 intracellular non−membrane−bounded organelle
1.1e−45 single−organism organelle organization
2.4e−43 cilium assembly
4.7e−43 microtubule−based process
1.9e−38 non−membrane−bounded organelle
2.0e−36 cell projection assembly
4.4e−34 cytoskeleton
1.4e−29 cellular component organization or biogenesis
8.1e−28 ciliary basal body
1.1e−26 cellular component organization
3.5e−25 organelle part
1.4e−24 axoneme
4.2e−24 smoothened signaling pathway
5.8e−22 supramolecular complex
6.7e−22 microtubule organizing center part
1.2e−21 centrosome
9.7e−20 organelle
2.2e−18 photoreceptor cell maintenance
1.3e−17 single−organism process
3.5e−16 single−organism cellular process
5.7e−16 microtubule cytoskeleton
6.3e−15 photoreceptor cell cilium
axoneme 3.0e−07
microtubule 1.1e−05
cilium 1.7e−05
microtubule cytoskeleton 1.4e−04
flagellum 2.5e−04
cilium morphogenesis 1.3e−03
microtubule−based flagellum 3.0e−03
microtubule−based movement 7.1e−03
cell projection 1.3e−02
ER to Golgi transport vesicle 1.7e−02
ER to Golgi transport vesicle membrane 1.7e−02
axoneme part 1.9e−02
iron ion binding 2.3e−02
MHC protein complex 3.1e−02
tetrapyrrole binding 3.5e−02
cell projection part 4.4e−02
cilium part 4.6e−02
MHC class II receptor activity 6.9e−02
protein folding 7.3e−02
microtubule basal body 1.2e−01
oxygen binding 1.5e−01
cellular component assembly involved in morphogenesis 2.2e−01
integral to lumenal side of endoplasmic reticulum membrane 2.9e−01
hair follicle development 3.3e−01
phenol−containing compound metabolic process 3.3e−01
integral to organelle membrane 3.5e−01
cobalt ion transport 3.5e−01
xenobiotic metabolic process 3.6e−01
cytoplasm 4.0e−01
intrinsic to organelle membrane 4.2e−01
1.2e−13 cilium
1.3e−05 ciliary part
1.0e−04 axoneme
1.0e−03 cilium organization
1.8e−03 lumenal side of endoplasmic reticulum membrane
2.7e−03 microtubule cytoskeleton
5.6e−03 cell projection
9.3e−03 microtubule−based movement
1.1e−02 MHC protein complex
1.7e−02 ER to Golgi transport vesicle membrane
2.4e−02 iron ion binding
3.2e−02 tetrapyrrole binding
4.1e−02 single−organism process
6.6e−02 MHC class II receptor activity
1.6e−01 oxygen binding
1.7e−01 ciliary basal body
1.9e−01 ER to Golgi transport vesicle
2.1e−01 sulfation
2.6e−01 cell projection part
2.6e−01 microtubule
2.7e−01 integral component of plasma membrane
3.0e−01 integral component of lumenal side of endoplasmic reticulum membrane
3.0e−01 protein folding
3.5e−01 intrinsic component of plasma membrane
3.5e−01 xenobiotic metabolic process
3.7e−01 cobalt ion transport
4.8e−01 cilium assembly
5.5e−01 phenol−containing compound metabolic process
6.9e−01 cytoskeleton
9.9e−01 regulation of cytokine production involved in immune response
2012−12−01 vs.old GO (2012) & new gene annotations (2017)
cell projection 2.0e−92
cell projection part 4.4e−76
cilium 6.1e−72
microtubule organizing center 3.4e−61
cilium part 4.7e−60
axoneme 1.0e−59
cytoskeletal part 3.0e−59
cilium morphogenesis 4.9e−55
microtubule−based process 1.0e−49
cellular component assembly involved in morphogenesis 4.2e−48
non−membrane−bounded organelle 2.5e−43
intracellular non−membrane−bounded organelle 3.9e−43
cell projection organization 6.1e−32
microtubule basal body 7.2e−29
cytoskeleton 8.7e−29
cilium assembly 1.6e−27
cell projection assembly 4.1e−27
cellular component organization or biogenesis 6.7e−27
multi−organism process 7.4e−26
organelle part 1.0e−24
cellular developmental process 2.3e−24
smoothened signaling pathway 3.0e−24
microtubule organizing center part 1.3e−22
centrosome 2.9e−22
developmental process 3.3e−20
microtubule cytoskeleton 4.2e−20
axoneme part 1.4e−18
cellular component morphogenesis 2.6e−17
flagellum 5.0e−15
macromolecular complex 1.2e−14
2.1e−129 cell projection
7.0e−105 microtubule−based process
1.5e−103 cell projection part
5.8e−86 axoneme
2.9e−80 microtubule organizing center
7.1e−77 cilium part
6.3e−76 cytoskeletal part
3.7e−75 cilium
1.5e−64 cilium morphogenesis
5.7e−52 smoothened signaling pathway
1.6e−51 axoneme part
6.6e−48 cellular component organization or biogenesis
7.7e−48 non−membrane−bounded organelle
2.0e−47 cell projection organization
7.8e−45 multi−organism process
3.2e−44 intracellular non−membrane−bounded organelle
3.9e−43 microtubule organizing center part
9.7e−42 centrosome
1.5e−36 cellular component morphogenesis
3.4e−36 microtubule−based movement
4.7e−36 cellular developmental process
6.3e−36 cytoskeleton
2.8e−35 organelle part
3.4e−34 cellular component movement
3.9e−34 developmental process
6.5e−31 intraflagellar transport particle
5.7e−29 anatomical structure formation involved in morphogenesis
8.5e−29 microtubule basal body
4.8e−26 cell projection morphogenesis
2.4e−25 microtubule cytoskeleton
axoneme 3.0e−07
microtubule 1.1e−05
cilium 1.7e−05
microtubule cytoskeleton 1.4e−04
flagellum 2.5e−04
cilium morphogenesis 1.3e−03
microtubule−based flagellum 3.0e−03
microtubule−based movement 7.1e−03
cell projection 1.3e−02
ER to Golgi transport vesicle 1.7e−02
ER to Golgi transport vesicle membrane 1.7e−02
axoneme part 1.9e−02
iron ion binding 2.3e−02
MHC protein complex 3.1e−02
tetrapyrrole binding 3.5e−02
cell projection part 4.4e−02
cilium part 4.6e−02
MHC class II receptor activity 6.9e−02
protein folding 7.3e−02
microtubule basal body 1.2e−01
oxygen binding 1.5e−01
cellular component assembly involved in morphogenesis 2.2e−01
integral to lumenal side of endoplasmic reticulum membrane 2.9e−01
hair follicle development 3.3e−01
phenol−containing compound metabolic process 3.3e−01
integral to organelle membrane 3.5e−01
cobalt ion transport 3.5e−01
xenobiotic metabolic process 3.6e−01
cytoplasm 4.0e−01
intrinsic to organelle membrane 4.2e−01
5.9e−18 axoneme
3.8e−13 axoneme part
5.4e−13 cilium morphogenesis
6.1e−13 cilium
8.9e−11 microtubule cytoskeleton
1.8e−09 microtubule−based process
3.5e−09 axonemal dynein complex assembly
3.9e−09 intraflagellar transport particle
7.1e−09 intraflagellar transport particle B
3.8e−06 cell projection
5.4e−06 intraflagellar transport
9.8e−06 microtubule−based movement
3.5e−05 cytoskeleton−dependent intracellular transport
4.5e−05 cell projection part
5.4e−05 cytoskeleton
6.6e−05 microtubule
7.6e−05 centrosome
6.7e−04 cytoskeletal part
1.3e−03 MHC protein complex
3.0e−03 organic acid metabolic process
3.8e−03 ER to Golgi transport vesicle
3.8e−03 microtubule organizing center
4.7e−03 cilium part
7.5e−03 cell projection organization
1.3e−02 cytoplasm
2.3e−02 microtubule basal body
2.4e−02 cilium assembly
2.4e−02 carboxylic acid metabolic process
3.5e−02 smoothened signaling pathway
4.4e−02 peptide antigen binding
